# Proteome-wide analysis by liquid chromatography tandem mass spectrometry reveals the role of retinoic acid during adipogenesis in human bone mesenchymal stem cells

**DOI:** 10.7717/peerj.20846

**Published:** 2026-02-13

**Authors:** Jiaxin Peng, Siyu Chen, Shilei Nong, Yifan Chen, Zhenjie Wang, Tao Wang, Jun Cao

**Affiliations:** 1 Jiangxi Provincial Key Laboratory of Cell Precision Therapy, School of Basic Medical Sciences, Jiujiang University, Jiujiang, China; 2 Clinical Medical School of Jiujiang University, Jiujiang, China; 3 Key Laboratory of Translational Medicine, Jiujiang University, Jiujiang, China

**Keywords:** Human bone mesenchymal stem cells, Adipogenesis, Retinoic acid, LC-MS/MS, Proteomics

## Abstract

**Objective:**

Retinoic acid (RA), an active metabolite of vitamin A, may regulate adipogenesis and is associated with osteoporosis. To clarify the regulatory mechanism of RA in adipogenesis and its relationship with the occurrence and development of osteoporosis, we investigated the role of all-trans retinoic acid (ATRA) in protein expression profiling during human bone mesenchymal stem cells (hBMSCs) adipogenesis.

**Methods:**

Liquid chromatography tandem mass spectrometry (LC-MS/MS) was used to determine the protein profile, and raw data were analyzed against the UniProt database using MaxQuant with the Andromeda search engine. The Gene Ontology (GO) and Kyoto Encyclopedia of Genes and Genomes (KEGG) databases were used for functional annotation of differentially expressed proteins (DEPs). The interaction relationships of DEPs were assessed using the STRING database, and Cytoscape was used to visualize the protein interaction network.

**Results:**

A total of 5,611 proteins were identified by LC-MS/MS in 15 samples, of which 5,470 proteins showed quantifiable data. When treated with ATRA for seven and 14 days after adipogenic induction, 470 and 1,408 DEPs were upregulated and 508 and 1,345 DEPs were downregulated. Gene functional annotation of DEPs showed that ATRA upregulated classic signaling pathways, such as Wnt, Hippo, and MAPK, as well as cytoskeleton related pathways, including focal adhesion, extracellular matrix (ECM)-receptor interaction, and the regulation of actin cytoskeleton. ATRA downregulated many pathways related to metabolism, including the AMP-activated protein kinase (AMPK) and peroxisome proliferator activated receptor (PPAR) pathways, to repress adipocyte differentiation and lipid accumulation. Specifically, the inhibition of adipogenesis by ATRA was significantly attenuated when the Rho-associated protein kinase (ROCK) inhibitor Y27632 was used to block the regulation of actin cytoskeleton pathways.

**Conclusion:**

Our study suggests that ATRA downregulates metabolism-related pathways to inhibit the adipogenesis of hBMSCs by upregulating some classic signaling pathways and cytoskeleton-related pathways, indicating that ATRA may be a broad-spectrum metabolic inhibitor.

## Introduction

Adipocytes, a type of common connective tissue cell, play a critical role in maintaining energy balance through lipid storage and release as well as endocrine and paracrine regulation *via* adipokine secretion. Mature adipocytes are localized in various adipose tissues throughout the body, including white adipose tissue (WAT), brown adipose tissue (BAT), beige/brite adipose tissue, and bone marrow adipose tissue (BMAT). The adipocytes from different tissues exhibit distinct metabolic characteristics and physiological functions (Ghesmati et al., 2024; Suchacki et al., 2020). Specifically, BMAT adipocytes originate from bone mesenchymal stem cells (BMSCs) with multilineage differentiation potential for adipogenesis, osteogenesis, and chondrogenesis (Robert et al., 2020). Abnormal adipogenesis of BMSCs has been associated with diseases such as osteoporosis, an age-related condition characterized by low bone mass and structural degeneration of the bone tissue (Li et al., 2024; Rinotas et al., 2024; Tian, Lu & Meng, 2023).

Adipogenesis involves a series of complex cascades, including the sequential activation of several critical transcription factors CCAAT/enhancer-binding proteins *β* and *α* (C/EBP*β* and C/EBP*α*, respectively) and peroxisome proliferator-activated receptor*γ* (PPAR*γ*). C/EBP*β* is rapidly expressed in the early stages of adipogenesis and decreases during terminal differentiation. As an upstream transcription factor, C/EBP*β* subsequently induces the expression of C/EBP*α* and PPAR*γ* by binding to C/EBP regulatory elements. C/EBP*α* and PPAR*γ* further promote the expression of downstream adipogenic genes such as *FABP4*, *CD36*, and *FASN* (Ambele et al., 2020; Mota de Sá et al., 2017). Some signaling pathways, including transforming growth factor-beta (TGF*β*)/SMAD, wingless-type MMTV integration site (Wnt), Hedgehogs (Hh), Notch, regulation of actin cytoskeleton, *etc*., have been reported to influence cellular adipogenic differentiation (Badralmaa & Natarajan, 2025; James et al., 2010; Li & Wu, 2020; Potolitsyna et al., 2024; Yamaguchi et al., 2021).

Retinoic acid (RA), an active metabolite of vitamin A, plays a crucial role in embryonic development and organogenesis by influencing cell proliferation, differentiation, and metabolism (Wu et al., 2024). RA exists in various natural stereoisomeric forms such as all-trans retinoic acid (ATRA) and 9-cis-retinoic acid (9CRA).These isomers bind to retinoic acid receptors (RARs) or retinoid X receptors (RXRs) as ligands. ATRA binds to RARs with high affinity, whereas 9CRA is the primary ligand for RXRs. RARs and RXRs, each composed of *α*, *β*, and *γ* subtypes, belonging to the nuclear receptor superfamily (Petkovich & Chambon, 2022). Liganded receptors form RARs/RXRs heterodimers that bind to the retinoic acid response elements (RARE) of target genes and activate transcription as transcription factors (Pohl & Tomlinson, 2020).

RARs and RXRs show different effects on adipogenic differentiation. Some studies show RA suppresses adipogenic differentiation *via* RARs. Specifically, RA inhibits the transcriptional activity of C/EBP*β* through RARs in 3T3-L1 cells, thereby blocking the expression of downstream genes, *e.g.*, *PPARG* (Schwarz et al., 1997). This inhibition is associated with the blockage of C/EBP*β* phosphorylation at Thr188 (Ayala-Sumuano et al., 2016). However, other studies have reported contradictory results. For example, in high glucose (HG) conditions, RA enhances lipid accumulation and increases the expression of fatty acid synthase (FAS) and sterol regulatory element-binding protein (SREBP-1) (Abd Eldaim et al., 2017). Previous research indicates that RARs agonist ATRA exerts dose-dependent effects on adipogenesis, it promotes adipogenesis at low concentrations but inhibits it at high concentrations (Kim, Lee & Lee, 2019). Unlike RARs, RXRs promote adipogenic differentiation and regulate lipid metabolism by RXRs/PPAR*γ* heterodimers (Corrales, Vidal-Puig & Medina-Gómez, 2018).

In our previous study, we demonstrated that ATRA promotes early-stage adipogenesis in human bone mesenchymal stem cells (hBMSCs) by upregulating the expression of C/EBP*β* while inhibiting later stages *via* activation of the TGF-*β*/SMAD and Wnt/*β*-catenin pathways (Cao et al., 2017). To further understand the effects of RARs in adipogenesis at the proteomic level, in current study, we investigated the role of ATRA in hBMSCs adipogenesis using differential protein sequencing based on liquid chromatography tandem mass spectrometry (LC-MS/MS) and functional annotation analysis. Our findings indicate that the inhibitory effect of RA on adipogenesis involves the upregulation of some classic pathways and cytoskeleton-related pathways and subsequent downregulation of metabolic pathways.

## Materials and Methods

### hBMSCs culture and adipogenic induction

hBMSCs (Cyagen Biosciences, Guanzhou, China) were cultured in modified Eagle’s medium(MEM) supplemented with 10% (v/v) fetal bovine serum (FBS) (Gibco, Grand Island, NY, USA), 100 units/mL penicillin, and 100 µg/mL streptomycin in a humidified atmosphere containing 5% CO_2_ at 37 °C. Cells were grown to 80% confluence and passed in a 1:2 split ratio. The sixth passage of cells was used for further experiments. Upon reaching 100% confluency, cells were induced to differentiate in adipogenic induction medium containing *α*-MEM (Hyclone, Logan, UT, USA) with 10% FBS, 2 mM glutamine, 100 units/mL penicillin, 0.1 mg/mL streptomycin, 10 µg/mL insulin, 500 µM 3-isobutyl-1-methylxanthine (IBMX), 200 µM indomethacin, and 1 µM dexamethasone (Sigma-Aldrich, St. Louis, MO, USA). The induction medium was changed every 2–3 days. ATRA (Sigma-Aldrich, St. Louis, MO, USA) and Y27632 (MeilunBio, Dalian, China) were dissolved in dimethyl sulfoxide (DMSO) (Sigma-Aldrich, St. Louis, MO, USA) for this study, ensuring that the final concentration of DMSO did not exceed 0.1%(v/v) in any assays. The final working concentration of ATRA and Y27632 were 1 µM and 20 µM respectively. Induction and culture were performed *in vitro* according to the protocol described above, and cells at three time points (0 d, 7 d, and 14 d) were collected for subsequent treatment.

### LC-MS/MS detection

For the proteomic study, we prepared three biological replicates of five samples (hBMSCs, hBMSCs treated with adipogenic induction medium for 7 d or 14 d with or without 1 µM ATRA). The entire LC-MS/MS workflow includes protein extraction, enzymatic hydrolysis, tandem mass tags (TMT)-labelling, high performance liquid chromatography (HPLC) fractionation, mass spectrometry (MS) detection, raw data analysis and bioinformatics analysis.

One-step protein reduction and alkylation were processed with TCEP (tris (2-carboxyethyl) phosphine hydrochloride) and CAA (2- chloroacetamide), followed by trypsin digestion overnight. The digested peptides were desalted and then labeled with TMT-labelling reagents. The labeled peptides were then mixed and fractionated using high pH reverse phase chromatography into 15 fractions. Then the peptides were desalted and loaded onto a Q Exactive HF-X mass spectrometer coupled with an Easy-nLC 1200 system (both Thermo Scientific) and analyzed in data-dependent acquisition (DDA) mode (Li et al., 2020).

### Raw data analysis

Raw MS data were analyzed against the UniProt protein database using MaxQuant (V1.6.6) with the Andromeda search engine, and the search parameters were meticulously set to ensure accurate analysis of the protein data. Search results were filtered to maintain a false discovery rate (FDR) of 1% at both the protein and peptide levels. Proteins identified as decoy hits, contaminants, or site-specific modifications were excluded from further analyses. Principal component analysis (PCA) was performed to validate the reproducibility of the proteomic data, and the remaining proteins were subjected to differential expression analysis using the Diamond program in eggNOG-mapper software. Proteins with fold change (FC) ≥1.2 or FC ≤ 0.833 and a *p*-value ≤ 0.05 were classified as differentially expressed proteins (DEPs).

### Gene functional annotation of DEPs

Gene Ontology (GO) and Kyoto Encyclopedia of Genes and Genomes (KEGG) annotations of DEPs were performed using the GO and KEGG databases. GO annotation included three main categories: biological processes (BP), molecular functions (MF), and cellular components (CC). KEGG pathways included cellular processes, envionmental information processing, genetic information processing, metabolism, human diseases and organismal systems. STRING was used to evaluate the interaction relationships between DEPs, and Cytoscape software (version 3.10.3) was used to obtain the protein-protein interaction (PPI) network of DEPs.

### Oil Red O staining

Cells in six-well plates were washed with phosphate-buffered saline (PBS), and fixed with 4% formaldehyde in PBS at room temperature for 15 min, then stained with the Oil Red O working solution at room temperature for 30 min. Oil Red O working solution was prepared by mixing Oil Red O stock solution with distilled water in a ratio of 3:2. The cells were washed with PBS for three times, and imaged using the Olympus IX73 inverted microscope system. The staining was quantified by extracting the dye with isopropanol, and measuring the optical density (OD) at 540 nm.

### Statistical analyses

Statistical differences were evaluated using SPSS (version 27.0; SPSS Inc., Chicago, IL, USA), with significance set at *p* < 0.05. Significant DEPs were determined by the *p*-value from Student’s *t*-test and fold change thresholds, and Fisher’s exact test was performed to reveal significantly enriched GO terms and pathways.

## Results

### Overview of proteomic data from mass spectrometry

RARs agonist ATRA have shown a significant inhibition on adipogenesis of hBMSCs in our exprements, to clarify the impacts of RARs at the proteomic level, LC-MS/MS was employed to analyze protein expression profiles at 7 d and 14 d after induction with 1 µM ATRA. The selection of time points and ATRA concentrations was based on data from our previous study (Cao et al., 2017).

**Figure 1:**
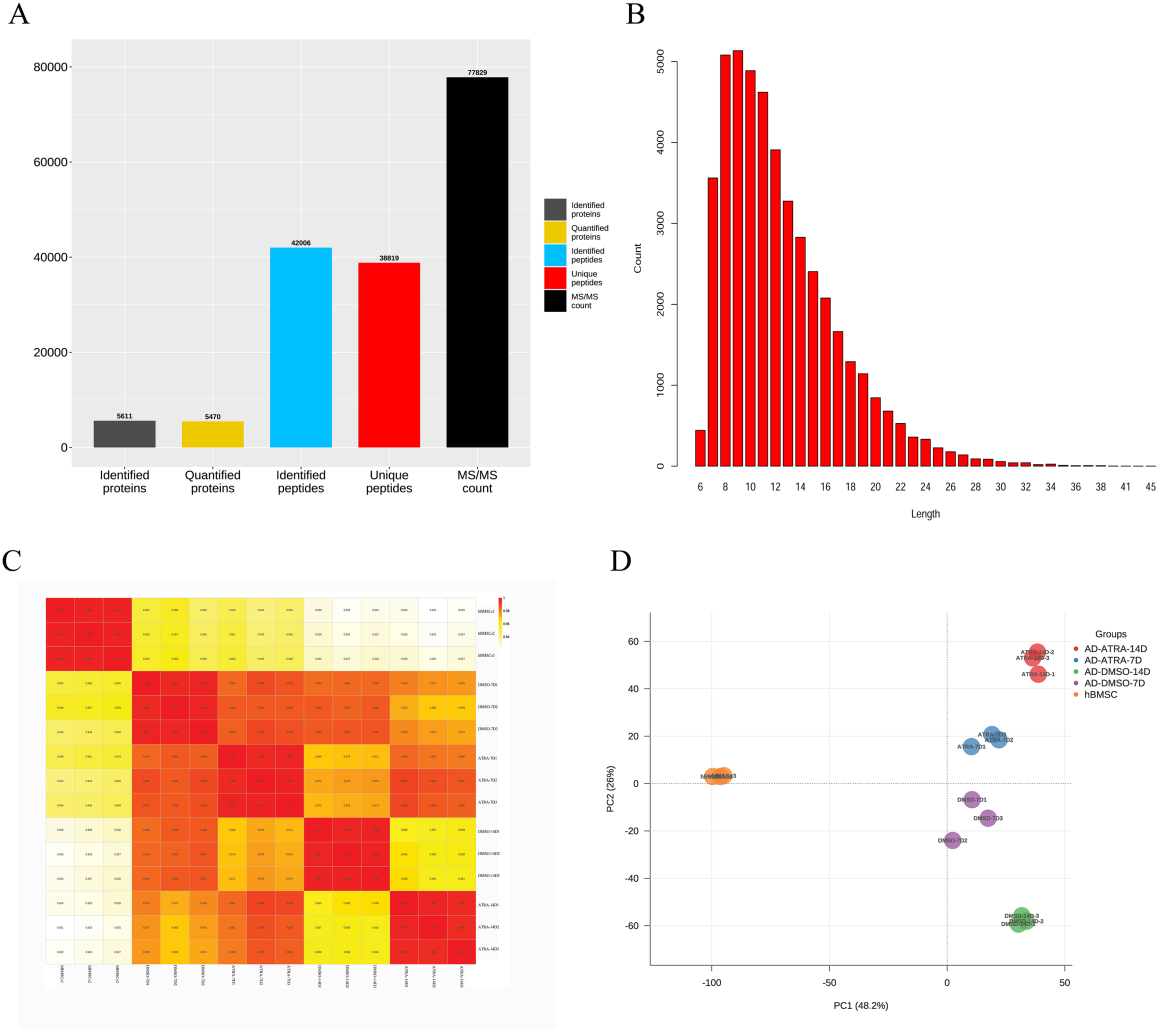
The quantitative correlation analysis and PCA analysis of proteins detected by LC-MS/MS. (A) The numbers of proteins detected by LC-MS/MS; (B) distribution of peptide lengths; (C) quantitative protein correlation coefficient distribution map, showing the quantitative correlation between the proteins contained in each sample, the deeper the red, the higher the correlation and repeatability; (D) principal component analysis (PCA) analysis of proteins, each point represents a sample, with the color indicating its group; the horizontal and vertical axes represent two group variables derived from dimensionality reduction analysis. Abbreviations: LC-MS/MS, Liquid chromatography tandem mass spectrometry; PCA, Principal component analysis.

A total of 5,611 proteins were identified by mass spectrometry in 15 samples. The search results were filtered using a 1% FDR at the protein and peptide levels. Entries of anti-library proteins, contaminating proteins, and proteins with only one modified peptide were excluded. Among them, 5,470 proteins had quantitative information for subsequent analysis. The identification, quantitative analysis, and peptide information of the proteins in the samples were subjected to comprehensive statistical analysis (Fig. 1A). A total of 5,470 proteins possessed quantifiable data and were selected for further analyses. The distribution of peptide lengths revealed that most peptides detected *via* MS ranged from seven to 27 amino acids in length (Fig. 1B). The coefficient of variation for the quantified proteins (Fig. 1C) demonstrated a high correlation between different samples, with a correlation coefficient exceeding 0.9, indicating a robust consistency in protein quantification across samples. PCA plots demonstrated distinct clustering within groups and a clear separation between groups based on differential protein expression, suggesting that the experimental design and execution were reliable (Fig. 1D).

### Analysis of DEPs

**Figure 2:**
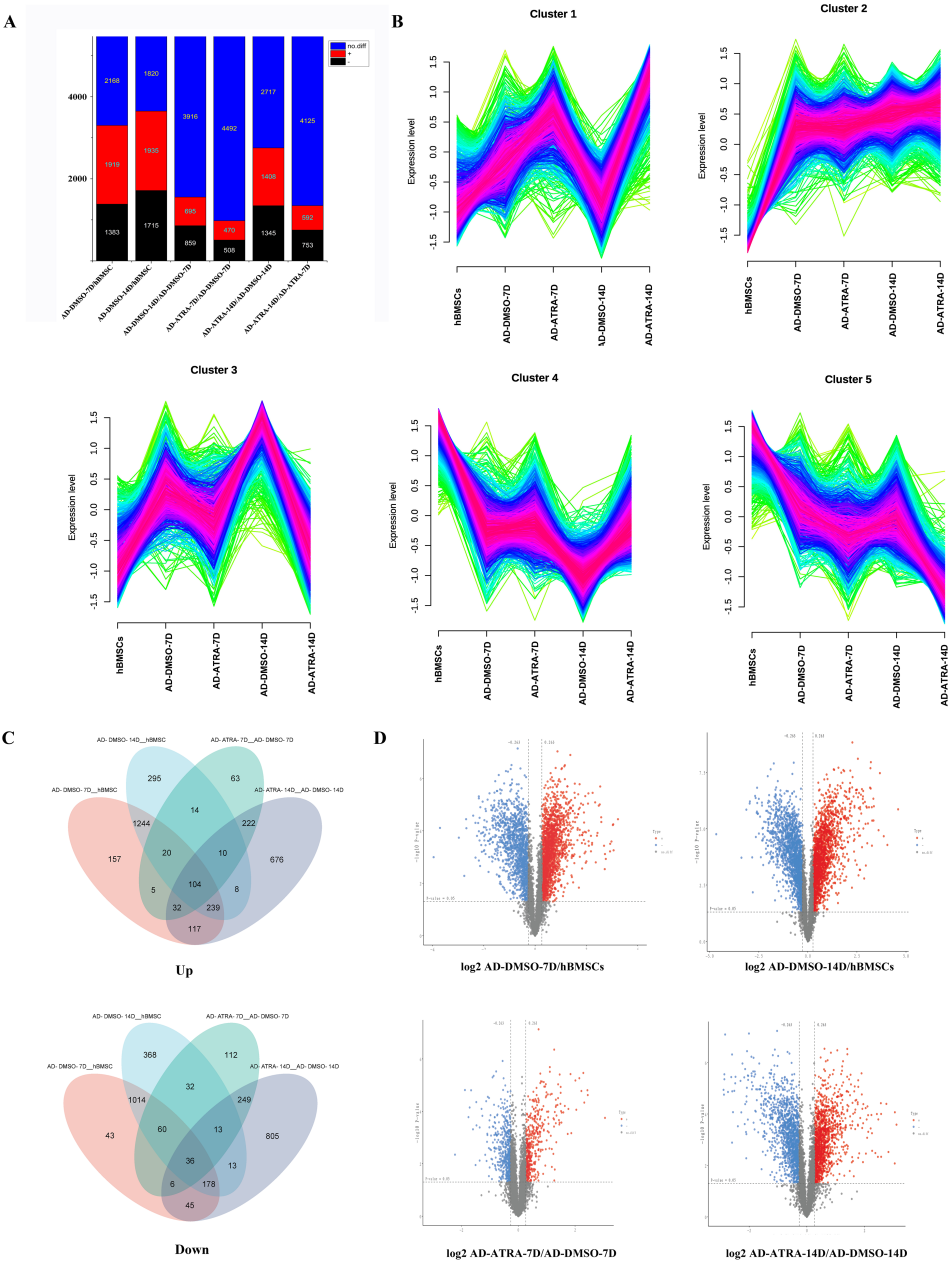
Identification of DEPs in the four comparisons. (A) Statistical map of differential proteins shows the number of different proteins; (B) Mfuzz cluster analysis illustrates the changes in protein expression during adipogenic differentiation in hBMSCs development. The colors represent the match degrees between changes of proteins and the major changes of the clusters (red, blue and green indicating high, moderate and low match degrees respectively); (C) two Venn diagrams reveal overall DEPs by combining four DEPs sets; (D) volcano plots reveal the DEPs between each comparison. Abbreviations: DEPs, differentially expressed proteins; hBMSCs, human bone mesenchymal stem cells.

To determine the influence of ATRA on the adipogenesis of hBMSCs, the proteins with FC ≥ 1.2 or FC ≤ 0.833 and FDR < 0.05 were considered as the criteria for DEPs. In AD-DMSO-7D *vs.* hBMSCs, there were 3,302 DEPs, of which 1,919 were upregulated and 1,383 were downregulated. At AD-DMSO-14D *vs.* hBMSCs, there were 3,650 DEPs, of which 1,935 were upregulated and 1,715 were downregulated. At AD-ATRA-7D (adipogenic induction for 7 d with 1 µM ATRA) *vs.* AD-DMSO-7D (adipogenic induction for 7 d with 0.1% DMSO as vehicle control), there were 978 DEPs, of which 470 were upregulated and 508 were downregulated. When comparing the AD-ATRA-14D and AD-DMSO-14D groups, the number of DEPs was 2,753, of which 1,408 were upregulated and 1,345 were downregulated (Figs. 2A, 2D). The top 20 DEPs that were upregulated in the AD-ATRA-7D *vs.* AD-DMSO-7D cells and downregulated in the AD-DMSO-7D *vs.* hBMSCs are listed in Table 1. In addition, adipogenic marker proteins FABP4, LPL and ADIPOQ significantly increased in the adipogenic induction group but markedly decreased in the ATRA-treated group, consistent with the qPCR and Western blotting results from our previous studies, but the adipogenic transcription factors such as CEBPB, CEBPA and PPARG which had been detected using western blotting in our experiments were not detected by LC-MS/MS (Cao et al., 2017).

**Table 1:** The top 20 DEPs upregulated at AD-ATRA-7D *vs.* AD-DMSO-7D and downregulated at AD-DMSO-7D *vs.* hBMSCs.

		**AD-ATRA-7D *vs.* AD-DMSO-7D**			**AD-DMSO-7D *vs.* hBMSCs**		
**Accession**	**Gene**	**Fold change**	**Log2FC**	*P*-value	**Fold change**	**Log2FC**	*P*-value
P10909	CLU	4.440110546	2.150595596	5.45722E−06	0.532184841	−0.910000679	9.39162E−06
P21980	TGM2	4.03280788	2.011784678	6.648E−06	0.789918492	−0.340224299	0.000382109
P24592	IGFBP6	3.649094147	1.867538373	0.000681014	0.544696186	−0.87647633	0.005053938
O60669	SLC16A7	3.342093148	1.740751944	0.000729574	0.754326369	−0.406739237	0.017409059
O75911	DHRS3	2.641915829	1.401584503	3.13691E−05	0.705720835	−0.502830492	1.81232E−05
Q16647	PTGIS	2.605462476	1.381539477	5.72161E−05	0.812352297	−0.299822571	0.000221565
Q99541	PLIN2	2.518547109	1.332591717	5.9697E−05	0.679881574	−0.556644625	0.002030363
O14684	PTGES	2.499738026	1.321776907	3.59587E−05	0.668490519	−0.581020997	5.64159E−05
P17693	HLA-G	2.437524424	1.285416674	0.000106416	0.655964565	−0.608310213	0.00216218
P04921	GYPC	2.437488687	1.285395523	0.016620577	0.615782473	−0.699507291	0.036629997
Q9H799	CPLANE1	2.392780108	1.258687822	0.00038178	0.421590061	−1.246087237	0.000440482
P18827	SDC1	2.359917889	1.238736664	0.00018541	0.316299601	−1.660636358	6.23838E−05
Q8TCU6	PREX1	2.267140159	1.180873584	2.15733E−05	0.642031157	−0.639284785	1.68129E−06
P31431	SDC4	2.106293153	1.074706244	0.000634202	0.303691453	−1.719321789	0.000194064
P04439	HLA-A	2.084914464	1.059988197	0.000102333	0.631106552	−0.664044494	0.002161604
P22413	ENPP1	2.072120461	1.051107875	0.000169352	0.646657143	−0.628927096	0.000207954
Q8TD20	SLC2A12	2.013588466	1.009768858	0.000103443	0.431426453	−1.212813456	2.98516E−05
P50895	BCAM	1.998847126	0.999168138	0.001281475	0.647490251	−0.627069623	0.000228991
P35625	TIMP3	1.959179563	0.97024963	9.2529E−06	0.750047681	−0.414945784	0.000671778
Q96FN4	CPNE2	1.956516655	0.968287392	0.002791501	0.575111113	−0.798087378	0.003417003

By combining the four DEPs sets using a Venn diagram, 3,206 upregulated and 2,972 downregulated DEPs were identified (Fig. 2C). As shown in the figure, there were 1,244 proteins which only upregulated in adipogenic induction group (7D and 14D) and 222 proteins which only upregulated in ATRA-treated group (7D and 14D). In contrast, there were 1,014 proteins which only downregulated in adipogenic induction group and 249 proteins which only downregulated in ATRA-treated group. These proteins that only change in ATRA-treated group may be closely related to the effects of ATRA.

To further identify the proteins that play crucial roles in the adipogenic differentiation of hBMSCs, Mfuzz cluster analysis was performed to examine the expression trends of DEPs in the five groups (Fig. 2B). The DEPs were categorized into five different clusters based on their expression patterns. In particular, clusters three proteins exhibited upregulation in the induction control groups, but the upregulation was inhibited in the ATRA-treated groups, clusters four proteins exhibited downregulation in the induction control group, but the downregulation was inhibited in the ATRA-treated group.

### Gene ontology analysis of DEPs

**Figure 3:**
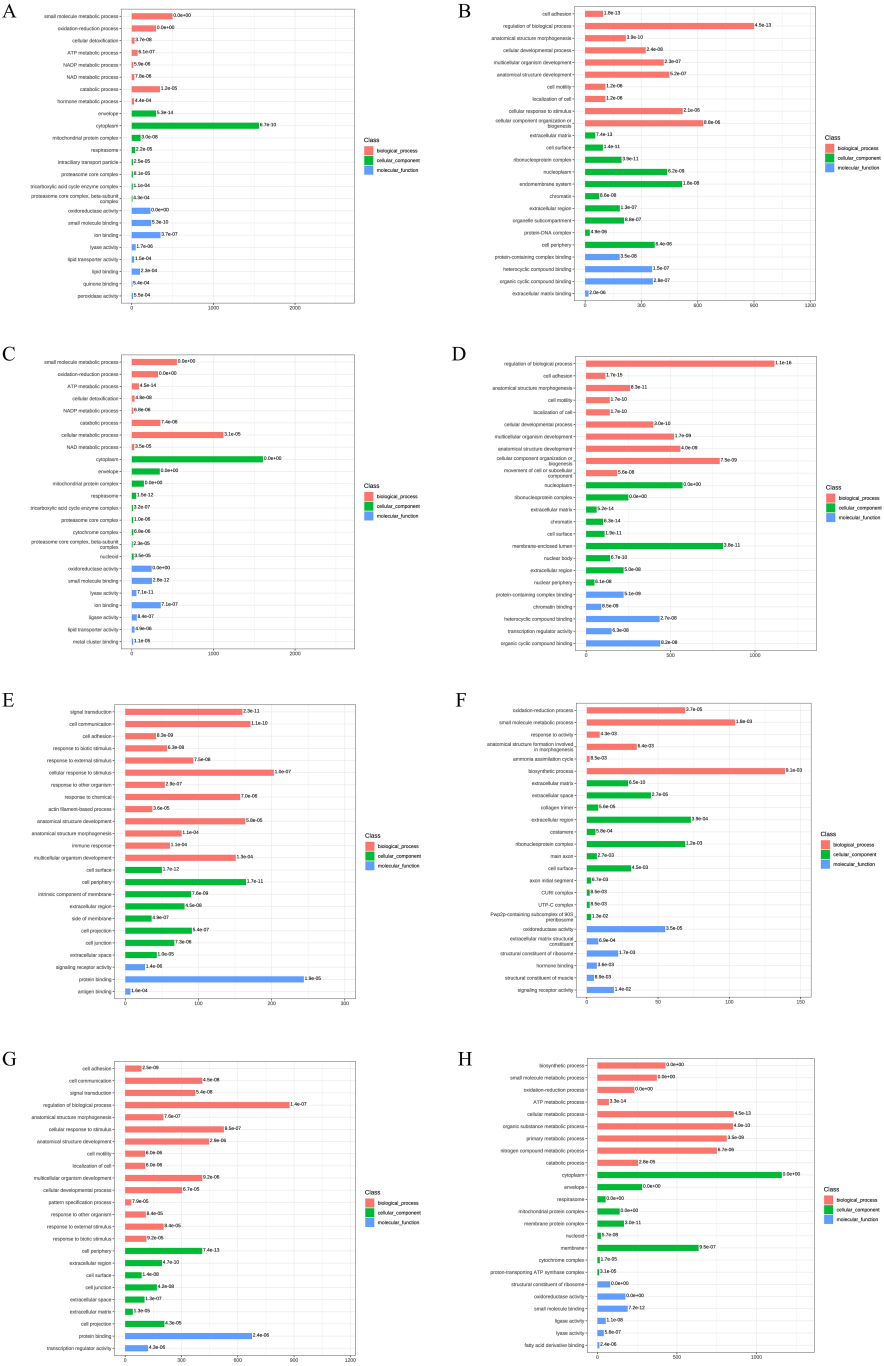
Gene Ontology analysis of DEPs. (A) The upregulated GO terms for AD-DMSO-7D *vs.* hBMSCs; (B) The downregulated GO terms for AD-DMSO-7D *vs.* hBMSCs; (C) The upregulated GO terms for AD-DMSO-14D *vs.* hBMSCs; (D) The downregulated GO terms for AD-DMSO-14D *vs.* hBMSCs; (E) The upregulated GO terms for AD-ATRA-7D *vs.* AD-DMSO-7D; (F) The downregulated GO terms for AD-ATRA-7D *vs.* AD-DMSO-7D; (G) The upregulated GO terms for AD-ATRA-14D *vs.* AD-DMSO-14D; (H) The downregulated GO terms for AD-ATRA-14D *vs.* AD-DMSO-14D. Abbreviations: GO, Gene Ontology; DMSO, dimethyl sulfoxide; ATRA, all-trans retinoic acid.

To reveal the functional properties and classifications of DEPs, we carried out the Gene Ontology (GO) analysis. The GO annotation results are summarized in Fig. 3. For upregulated DEPs from AD-DMSO-7D *vs.* hBMSCs, the enriched Biological Processes (BP) terms included small-molecule metabolic and oxidation- reduction processes, cellular detoxification,ATP and NADP metabolism. The Cellular Components (CC) terms comprised envelope, cytoplasm, mitochondrial protein complex, respirasome, and intraciliary transport particles. The Molecular Functions (MF) terms comprised oxidoreductase activity, small molecule binding, ion binding, lyase activity, lipid transporter activity, and lipid binding. For the downregulated DEPs in AD-DMSO-7D *vs.* hBMSCs, the enriched BP terms included cell adhesion, regulation of biological processes, anatomical structure morphogenesis, cellular developmental processes, and multicellular organism development. The CC terms consisted of extracellular matrix, cell surface, ribonucleoprotein complex, nucleoplasm, and endomembrane system. MF terms comprised protein-containing complex binding, heterocyclic compound binding, organic cyclic compound binding, extracellular matrix binding,and chromatin binding (Figs. 3A, 3B). The enriched GO terms for DEPs from AD-DMSO -14D *vs.* hBMSCs are shown in (Figs. 3C and 3D).

In contrast, for the upregulated DEPs in the AD-ATRA-7D *vs.* AD-DMSO-7D group, the enriched BP terms were signal transduction, cell communication, cell adhesion, and response to biotic stimulus. The CC terms included cell surface and periphery, intrinsic component of membrane, and extracellular region.The MF terms comprised signaling receptor activity and protein binding. For downregulated DEPs inthe AD-ATRA-7D *vs.* AD-DMSO-7D group, the enriched BP terms consisted of oxidation–reduction and small-molecule metabolic processes as well as response to activity. The CC terms consisted of extracellular matrix and space, collagen trimer, *etc.* The MF terms comprised oxidoreductase activity, extracellular matrix structural constituent, and structural constituent of ribosome.These terms indicated the influences of ATRA on adipogenesis (Figs. 3E, 3F). The enriched GO terms for the DEPs from the AD-ATRA-14D *vs.* AD-DMSO-14D groups are shown in (Figs. 3G and 3H).

### KEGG functional annotation and enrichment analysis of DEPs

**Figure 4:**
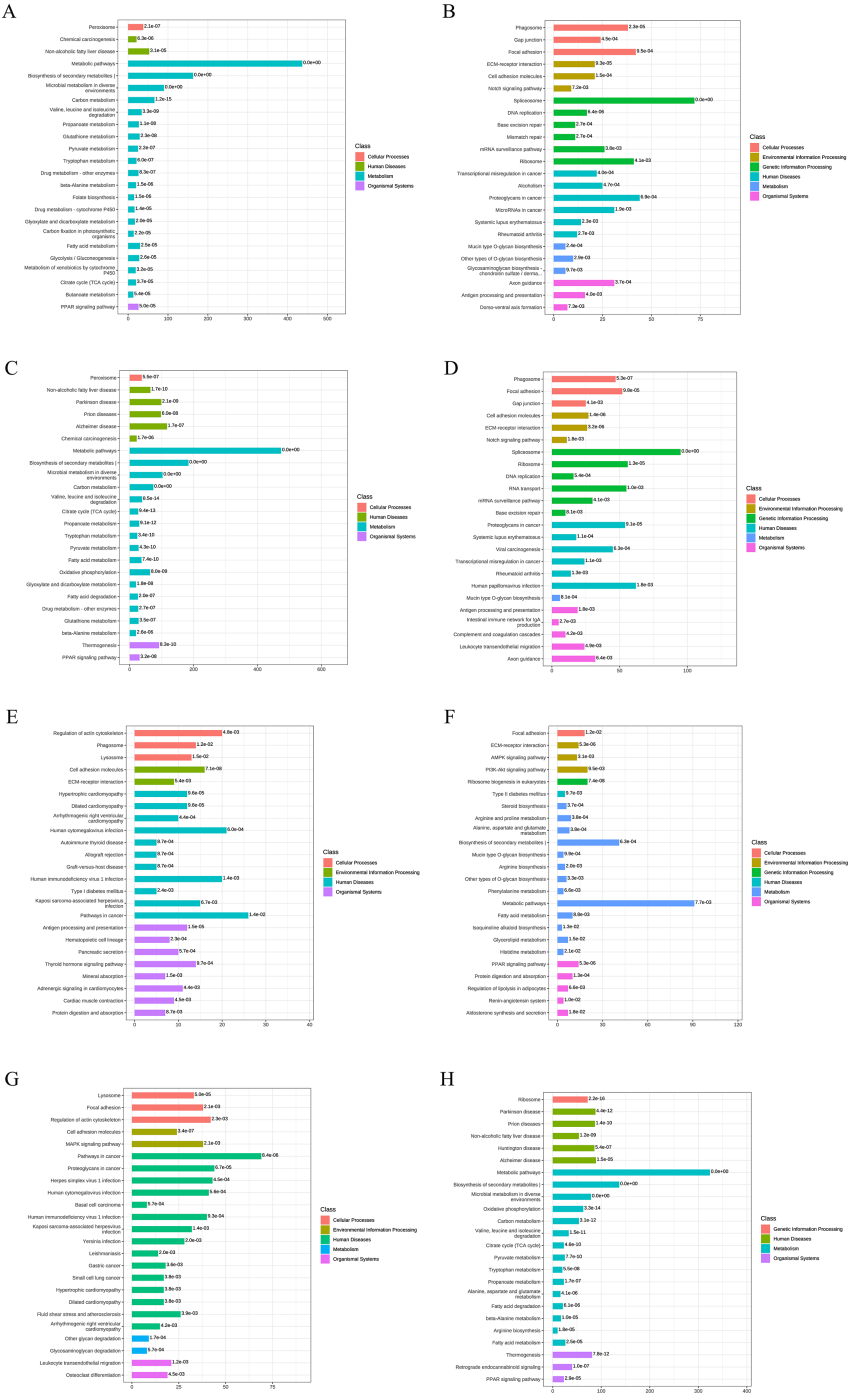
KEGG functional annotation and enrichment analysis of DEPs. (A) The upregulated pathways for AD-DMSO-7D *vs.* hBMSCs; (B) the downregulated pathways for AD-DMSO-7D *vs.* hBMSCs; (C) the upregulated pathways for AD-DMSO-14D *vs.* hBMSCs; (D) the downregulated pathways for AD-DMSO-14D *vs.* hBMSCs; (E) the upregulated pathways for AD-ATRA-7D *vs.* AD-DMSO-7D; (F) the downregulated pathways for AD-ATRA-7D *vs.* AD-DMSO-7D; (G) the upregulated pathways for AD-ATRA-14D *vs.* AD-DMSO-14D; (H) the downregulated pathways for AD-ATRA-14D *vs.* AD-DMSO-14D. Abbreviations: KEGG, Kyoto Encyclopedia of Genes and Genomes.

To better understand the functions of the identified DEPs in physiological and pathological processes and to determine the metabolic and signaling pathways in which they are involved, we carried out KEEG pathway analysis of the DEPs. At AD-DMSO-7D *vs.* hBMSCs, the upregulated DEPs were significantly enriched in 96 pathways respectively (*p* < 0.05), including peroxisome, calcium signaling pathway, Janus kinase=-signal transducer and activator of transcription protein= (JAK-STAT) signaling pathway, AMP-activated protein kinase (AMPK) signaling pathway, metabolic pathways, biosynthesis of secondary metabolites, microbial metabolism in diverse environments, carbon metabolism, valine, leucine, and isoleucine degradation, pyruvate metabolism, fatty acid metabolism, glycolysis/gluconeogenesis, citrate cycle, and peroxisome proliferator–activated receptor (PPAR) signaling pathway. The downregulated DEPs were significantly enriched in 43 pathways (*p* < 0.05), including phagosome, gap junction, focal adhesion,regulation of actin cytoskeleton, extracellular matrix (ECM) -receptor interaction,cell adhesion molecules, Notch signaling pathway, Rap1 signaling pathway, and spliceosome (Figs. 4A, 4B). The enriched pathways for DEPs from the AD-DMSO-14D *vs.* hBMSCs group were similar to those of the AD-DMSO-7D *vs.* hBMSCs group (Figs. 4C, 4D).

In contrast, at AD-ATRA-7D *vs.* AD-DMSO-7D, the upregulated DEPs were significantly enriched in 37 pathways, including regulation of actin cytoskeleton, phagosome, lysosome, cell adhesion molecules, extracellular matrix (ECM)-receptor interaction, Rap1 and cGMP-PKG signaling pathways, antigen processing and presentation, hematopoietic cell lineage, thyroid hormone signaling pathway, and mineral absorption. The downregulated DEPs were significantly enriched in 31 pathways, including Focal adhesion, ECM-receptor interaction, AMPK and PI3K-Akt signaling pathways, steroid biosynthesis, arginine and proline metabolism, metabolic pathways,biosynthesis of secondary metabolites, fatty acid metabolism, and PPAR signaling pathway (Figs. 4E, 4F). In the AD-ATRA-14D *vs.* AD-DMSO-14D comparison, upregulated and downregulated DEPs were significantly enriched in 55 and 64 pathways, respectively (Figs. 4G, 4H). In particular, ABC transporter as well as MAPK, TNF, Hippo, Wnt, and Notch signaling pathways were upregulated in the AD-ATRA-14D group.

### PPI networks indicated a possible connection between actin cytoskeleton and PPAR pathway

**Figure 5:**
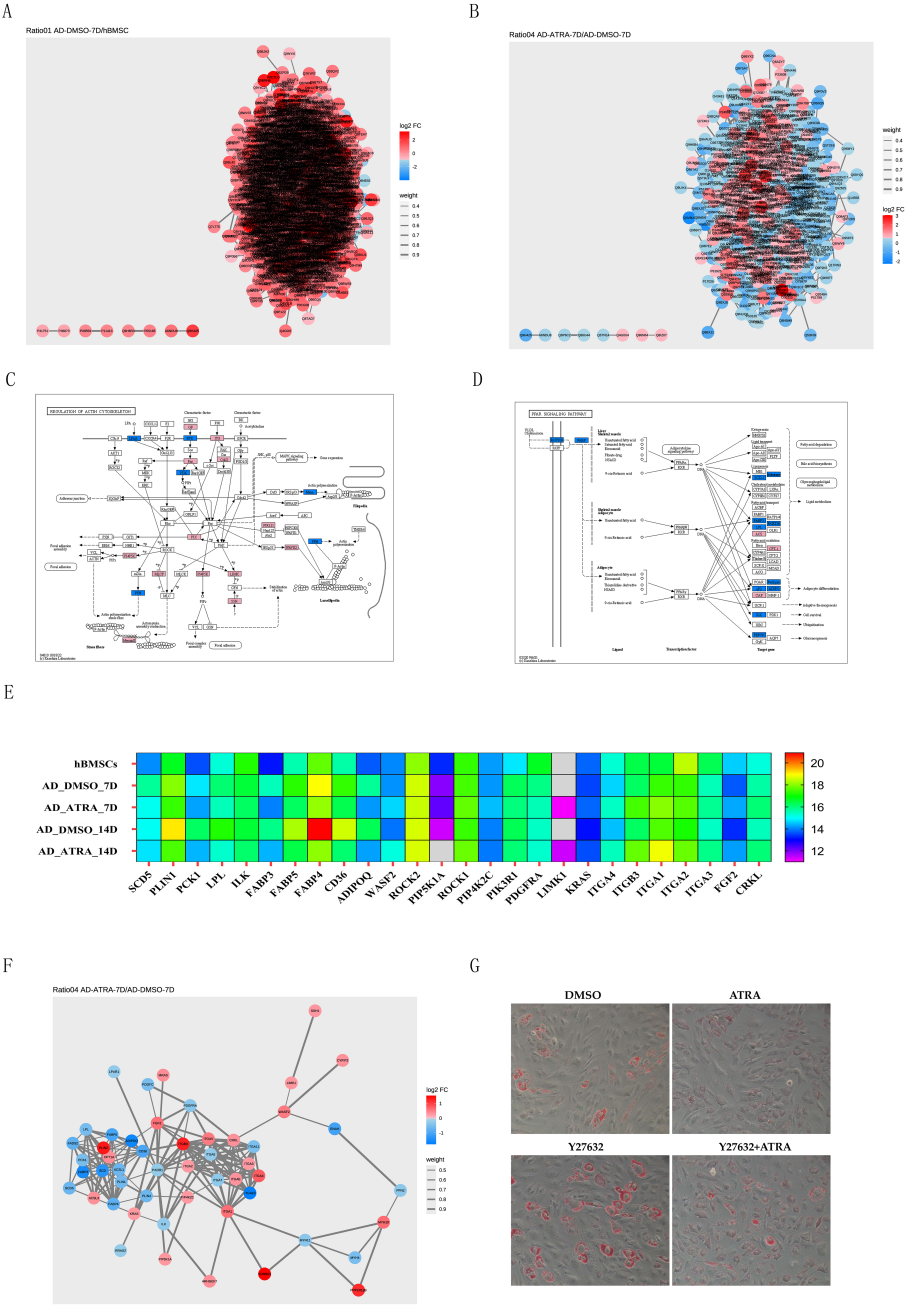
PPI networks implicated the adipogenesis inhibition of ATRA may be related to upregulation of the actin cytoskeleton pathway. (A) PPI networks comparing AD-DMSO-7D *vs.* hBMSCs, the colors of nodes indicate the expression level of the protein, where a gradient from red to blue shows a change in log_2_FC; (B) PPI networks comparing AD-ATRA-7D *vs.* AD-DMSO-7D; (C) KEGG diagram of the regulation of actin cytoskeleton pathway; (D) KEGG diagram of the PPAR signaling pathway; (E) heatmap of protein expression from the regulation of actin cytoskeleton and PPAR pathway; (F) PPI network between the regulation of actin cytoskeleton and PPAR pathway; (G) oil red O staining for Y27632 on adipogenesis of hBMSCs. Cells were treated with 1 µM ATRA and 20 µM Y27632 for seven days, then were determined by Oil Red O staining. The images were captured at 20× magnification. Abbreviations: PPI, protein-protein interaction; PPAR, peroxisome proliferator activated receptor.

PPI networks of DEPs from the AD-DMSO-7D *vs.* hBMSCs and AD-ATRA-7D *vs.* AD-DMSO-7D groups were constructed. Interactive relationships between different proteins were assessed using the STRING database. Network analysis was subsequently performed on the acquired data using Cytoscape software to visualize the protein interactions, where nodes represented individual proteins and edges denoted the interactions between them; the darker the color, the higher the degree value, indicating the importance or influence of the protein (Figs. 5A, 5B). We then chose the PPAR signaling pathway from the pathways downregulated by ATRA and the regulation of the actin cytoskeleton pathway from the pathways upregulated by ATRA and analyzed the interactions between these two pathways. Protein expression levels are displayed in the KEGG diagram and heatmap of protein expression (Figs. 5C, 5E). A PPI network between the regulation of actin cytoskeleton and the PPAR pathway was constructed (Fig. 5F). To verify whether ATRA (1 µM) inhibits the PPAR signaling pathway through the regulation of actin cytoskeleton pathway, we utilized the Rho-associated protein kinase (ROCK) inhibitor Y27632 (20 µM) to block the activity of ROCK. Oil Red O staining showed that ATRA-induced inhibition of adipogenesis was significantly attenuated by Y27632 treatment (Fig. 5G).

## Discussion

Numerous studies have demonstrated that RA plays a crucial role in regulating adipogenesis and is thought to be associated with osteoporosis (Lerner, 2024; Malibary, 2023). Our previous studies show that RA receptors RARs and RXRs play different roles on adipogenesis. RARs agonists ATRA inhibited the adipogenesis in the late stage but RXRs agonists SR11237 significantly promoted adipogenesis in hBMSCs (Cao et al., 2017). So far, there have been no reports of the impacts of RA on adipogenesis from a proteomic perspective, which is particularly important for further understanding the regulatory mechanism of RA. Therefore, we investigated the effects of RARs agonist ATRA on proteomic changes that occur during the differentiation of hBMSCs into adipocytes.

The data revealed that 470 and 508 proteins were up- and downregulated on day 7, respectively, whereas 1,408 and 1,345 proteins were up- and downregulated on day 14, respectively, in the ATRA-treated groups (AD-ATRA-7D and AD-ATRA-14D). PPAR signaling pathway protein (FABP4, LPL and ADIPOQ) significantly increased in the adipogenic induction group but markedly decreased in the ATRA-treated group. On the contrary, TGF*β* signling pathway protein SMAD3 and Wnt pathway protein CTNNB1, significantly decreased in the adipogenic induction group but markedly increased in the ATRA-treated group, which were consistent with western blotting results in our previous research, indicating the reliability of current LC-MS/MS detection (Cao et al., 2017).

GO annotation indicated that ATRA upregulated some biological processes, such as signal transduction, cell communication, cell adhesion, and regulation of biological processes, which were downregulated in the adipogenic control group, whereas other biological processes, including small molecule metabolic processes, oxidation–reduction, biosynthetic and cellular metabolic processes, were downregulated when ATRA was treated for 7 d but upregulated when adipogenesis was induced for 7d. Furthermore, ATP, NADP, NAD, and NADH metabolic processes as well as catabolic process were downregulated after ATRA treatment for 14 d. These findings suggest that some biological processes such as signal transduction, cell communication, and cell adhesion maybe negative regulatory factors for adipogenic differentiation. ATRA upregulates these processes, thus inhibiting metabolic processes, and the inhibition of metabolism becomes stronger with time, providing new evidence that ATRA inhibits adipogenesis. For example, cell adhesion and ECM are involved in adipogenesis and may be critical targets for RA.

Additionally, we found that ATRA inhibited biomineral tissue development when treated for 7d with HACD1, ALPL, FOXO1, and STIM1. These proteins participate in bone formation, for example, FOXO1, a transcription factor with forkhead box, is upregulated during bone formation and promotes the osteogenesis and formation of mineralizing nodules (Siqueira et al., 2011). Stim1 is a endoplasmic reticulum (ER) Ca^2+^ sensor protein and involves the maintenance of calcium homeostasis by store-operated Ca^2+^ entry (SOCE) (Chen et al., 2021b). Stim1 increases calcium deposits and induces the expression of the key osteogenic transcription factors Runx2 and type I collagen, promoting mineralized matrix formation (Chen et al., 2018b).

The role of RA in osteoporosis is still unclear. Numerous studies show that ATRA increases osteogenesis and inhibits adipogenesis, which promote a switch between osteogenesis and adipogenesis, but the excessive intake and accumulation of vitamin A in the organism is associated with a reduced bone mineral density. ATRA induces the expression of RANKL to enhance bone resorption in osteoclasts and inhibits the bone form in osteoblasts, which is widely used in the production of animal models of osteoporosis (Lerner, 2024). Current data revealed a new possible mechanism by which ATRA is involved in the occurrence and development of osteoporosis.

KEGG pathway analysis showed that ATRA upregulated some classic signaling pathways, such as Wnt, Hippo, MAPK, TNF, cGMP-PKG, Rap1, and Notch, whereas Rap1 and Notch were downregulated in the control groups. Our previous study has shown that Wnt pathway is involved in the inhibition of ATRA on adipogenesis, ATRA blocks the function of C/EBP*β* and PPAR*γ* by upregulating the expression of Wnt2B and *β*-catenin in the later stages of adipogenesis (Cao et al., 2017). The Hippo signaling pathway plays a central role in the regulation of cellular proliferation and differentiation. Yes-associated protein (YAP), a downstream effector protein in the Hippo pathway, may enhance the osteogenic differentiation of human MSCs and suppress adipocyte differentiation (Lorthongpanich et al., 2019). Connective tissue growth factor (CTGF) is a target gene of YAP; YAP1 interacts with transcription factor TEA domain 1 (TEAD1) to regulate the expression of CTGF (Roy et al., 2024). CTGF inhibits adipocyte differentiation (Tan et al., 2008). In this study, MS analysis showed that the expression levels of the Hippo pathway members, TEAD1 and CTGF, were elevated after ATRA treatment, indicating that ATRA is likely to inhibit the adipogenic differentiation of hBMSCs through YAP/TEAD1/CTGF. MAPK (Perumal et al., 2023), TNF (Ma et al., 2020), cGMP-PKG (Engin, 2024), Rap1 (Kim et al., 2010), and Notch (Wang et al., 2024) have been reported to be involved in adipogenesis. However, the relationships between ATRA and key adipogenic pathways remain unclear.

ATRA also upregulated cytoskeleton- and ECM-related pathways, including regulation of the actin cytoskeleton, focal adhesion, ECM-receptor interaction, and cell adhesion molecules, which were downregulated in the control group. The cytoskeleton and ECM participate in the regulation of adipogenesis by mechanotransduction; for example, ECM stiffness influences the activity of YAP, a key mechanosensor, to modulate adipocyte differentiation and lipid accumulation (Dong & Jin, 2024). Focal adhesion, the main hub for cell–matrix interactions, may transfer mechanical cues from the ECM to cellular cytoskeleton, regulating MSC differentiation (Raman et al., 2022).

In contrast, many pathways related to material and energy metabolism were downregulated by ATRA, including metabolic pathways, valine, leucine, and isoleucine degradation, fatty acid metabolism, glycolysis/gluconeogenesis, oxidative phosphorylation, and PPAR signaling pathways, ranging from amino acid metabolism, carbohydrate metabolism, and lipid metabolism to energy metabolism. These data suggested that ATRA functions as an extensive metabolic inhibitor. In particular, ATRA downregulates the AMPK signaling pathway, which is upregulated upon adipogenic induction. AMPK is widely regarded as a conserved master regulator of energy homeostasis that inhibits adipogenesis and lipid accumulation (Bustraan et al., 2024). However, the role of AMPK in adipogenesis is controversial, AMPK*β*2 has been reported to upregulate and promote adipogenesis in 3T3-L1 adipocytes (Katwan et al., 2019). In the present study, AMPK subunit proteins encoded by *PRKAA1*, *PRKAB1*, *PRKAG1*, and *PRKAG2* were upregulated under adipogenic induction, suggesting that the AMPK pathway may also have a positive regulatory effect on adipogenesis of hBMSCs, and the inhibition of AMPK pathway perhaps be one of the mechanisms by which it inhibits adipogenic differentiation. It should be noted that ATRA suppressed the expression of the AMPK gamma 1 subunit but promoted the expression of the alpha 1 subunit in this study; therefore, the effects of ATRA on AMPK require further investigation.

We further analyzed the interaction between regulation of the actin cytoskeleton pathway (ITGAV, FGF2, ITGB3, ITGA1, ITGA2, ITGA3, PIK3R1, KRAS, LIMK1, CRKL,and WASF2) and the PPAR signaling pathway (ADIPOQ, LPL, SCD, CD36, PCK1, FABP4, and PLIN1). The PPAR signaling pathway is essential for the initiation of adipogenesis. PPAR*γ* is a key transcription factor that regulates the expression of adipogenic marker genes, such as *FABP4*, *LPL*, and *ADIPOQ*, involved in adipogenic differentiation and is thought to function as a master switch for adipocyte specificity in acquiring adipocyte phenotypes.

The regulation of actin cytoskeleton pathway plays apivotal role in controlling cellular processes such as muscle contraction, cell signaling, and cell junction formation, as well as in modulating gene expression. It is also involved in the regulation of adipogenesis. Integrins are a type of transmembrane receptors, may act as mechanosensors to regulate the cytoskeleton-ECM interactions. In this study, ATRA upregulated the expression of the integrins such as ITGAV, FGF2, ITGB3, ITGA1, ITGA2 and ITGA3. It has been shown that ITGAV may interact with fibronectin to inhibit the terminal adipocyte differentiation (Uetaki et al., 2022). In another study, blockades of ITGA2, ITGA3 and ITGAV contribute to the adipogenic differentiation of human adipose-derived stem cells (Li et al., 2025). Therefore, ATRA is likely to inhibit adipogenic differentiation by upregulating the expression of integrin.

Actin exists in two primary forms, globular (G-actin) and filamentous (F-actin). F-actin is considered the foundation of the cytoskeleton (Khan et al., 2020). It has been demonstrated that actin cytoskeleton is dissembled during adipocyte differentiation in human stromal stem cells. The differentiation was inhibited by stabilizing F-actin and reducing G-actin levels, which is associated with changes in RhoA-ROCK- LIMK-CFL1 pathway (Chen et al., 2018a). ROCK, a downstream effector of the small GTPase Rho, may induce stressed actin filament formation, regulate actin cytoskeletal contractility, and mediate mechanophysical interactions between cells and ECM (Bouzid et al., 2022).

In the present study, the actin cytoskeleton pathway was upregulated and the PPAR signaling pathway was downregulated in the ATRA-treated group, indicating that the regulation of the actin cytoskeleton pathway probably inhibits the PPAR signaling pathway. Earlier studies have addressed that ROCK signaling suppresses adipogenesis by controlling PPAR*γ* expression and actin organization in adipocytes (Ji et al., 2017). The ROCK inhibitor, Y27632, suppresses LIMK activation, enhances adipogenic differentiation, and functions as a switch-like regulator to modulate hBMCs (Hyväri et al., 2018). Here,we provide useful information that Y27632 promotes adipogenesis and reverses the inhibitory effect of ATRA on cell differentiation, indicating that ATRA inhibition maybe related to the activation of ROCK and upregulation of the actin cytoskeleton pathway. However, the detailed molecular mechanisms require further exploration.

## Conclusion

In conclusion, our study for the first time describes the role of ATRA in protein expression profiling using TMT-labeled LC-MS/MS during adipogenesis in hBMSCs and demonstrates that inhibition of adipogenesis by ATRA involves upregulation of some classic signaling pathways and cytoskeleton-related pathways and subsequent downregulation of metabolic pathways, providing new insights into the molecular mechanisms of RA-mediated regulation of adipogenesis in hBMSCs. However, due to the limitation of MS resolution, some low-abundance upstream signal proteins including transcription factors such as PPAR*γ* and C/EBP*α*, which were detected significant decrease by ATRA during adipogenesis using western blotting in our previous studies, were not detected in this study, directly affecting the integrity of the results. This is a follow-up problem that must be addressed in future studies.

The mass spectrometry proteomics data have been deposited to the ProteomeXchange Consortium (https://proteomecentral.proteomexchange.org) *via* the iProX partner repository (Ma et al., 2019; Chen et al., 2021a) with the dataset identifier PXD065247.

## Additional Information and Declarations

### Data Availability

The following information was supplied regarding data availability: The mass spectrometry proteomics data are available at the ProteomeXchange Consortium: PXD065247.
